# Pattern of Infecting Microorganisms and Their Susceptibility to Antimicrobial Drugs in Patients with Diabetic Foot Infections in a Tertiary Care Hospital in Karachi, Pakistan

**DOI:** 10.7759/cureus.2872

**Published:** 2018-06-25

**Authors:** Ghulam M Kaimkhani, Adeel A Siddiqui, Nusrat Rasheed, Mohammad Irfan Rajput, Jagdesh Kumar, Mohammad Hassan Khan, Shahzadi Nisar, Sheema Mustafa, Uzair Yaqoob

**Affiliations:** 1 Orthopedic Surgery, Dow University of Health Sciences, Karachi, PAK; 2 Orthopaedic Surgery, Dow University of Health Sciences, Karachi, PAK; 3 Orthopaedic Surgery, Dow University of Health Sciences, Karachi , PAK; 4 Surgery, Jinnah Postgraduate Medical Centre, Karachi, PAK

**Keywords:** diabetic foot ulcer, diabetic osteomyelitis, polymicrobial infection

## Abstract

Diabetes mellitus is a universal health problem, with its prevalence in Pakistan making it among the top 10 countries in the world. Approximately 13.9 million people in Pakistan will have developed diabetes by 2030. Diabetic foot ulcer (DFU) is one of the more serious complications of diabetes. If not treated properly, patients may develop diabetic foot osteomyelitis leading to gangrene and amputation. These infections are usually polymicrobial, with Staphylococcus aureus (S. aureus), Proteus, Pseudomonas, and Escherichia coli (E. coli) being among the more common organisms isolated from DFU. This survey of patients with DFU in a tertiary hospital in Karachi, Pakistan found 68.5% of patients had peripheral neuropathy, 57% had chronic osteomyelitis, and 37% and 49% had Wagner grades 2 and 3, respectively. Infections were polymicrobial in 83% of patients, E. coli was isolated from 63%, and S. aureus from 58%. Of the isolated organisms, 95% were sensitive to meropenem and 81% to linezolid.

## Introduction

Diabetes mellitus is a universal health problem, affecting about 171 million people worldwide in 2000 and is estimated to affect 366 million people by 2030. The prevalence of diabetes in Pakistan is particularly high, making it among the top 10 countries in the world. Diabetes affected 5.2 million Pakistanis in 2000, but will likely affect approximately 13.9 million in 2030 [1].

Diabetic foot ulcer (DFU) is one of the most serious complications of diabetes, with a lifetime risk of developing foot infections ranging from 19% to 34% [2]. DFU frequently occurs in patients who do not take care of their feet. DFU initially presents as a superficial infection of soft tissues and bone associated with signs of inflammation and/or purulent discharge. Predisposing factors are peripheral neuropathy, small vessels angiopathy and impaired immune system [3]. If not treated properly, 44% to 68% of these patients with DFU may develop diabetic foot osteomyelitis, leading to gangrene and amputation [4]. These infections are usually polymicrobial, with Staphylococcus aureus, Proteus, Pseudomonas, and Escherichia coli being among the common organisms isolated from DFU. Methicillin-resistant S. aureus (MRSA) is present in 10% to 32% of diabetic foot infections and is associated with higher rates of treatment failure and foot amputation [3, 5]. Diabetes is the leading cause of lower limb amputation, with the risk of amputation being 15 to 40 times higher in patients with diabetes compared to those without diabetes [6]. In the United States (US), more than half of lower limb amputations are in diabetic patients [7]. The amputation rate is also high in Pakistan (21% to 48%) due to the improper initial management of foot ulcers, poor glycemic control, and patient noncompliance [8]. Most amputations can be avoided if DFU is diagnosed early and treated aggressively with wound debridement and appropriate antibiotics [8].

Microbial organisms isolated from patients with DFU can vary. Single organisms, such as S. aureus, are usually isolated from mild infections, whereas polymicrobial organisms, including Gram-positive cocci (such as S. aureus, S. epidermis, and enterococci), Gram-negative bacilli (such as Pseudomonas spp., E. coli, and Enterobacter spp), and aerobes (such as Bacteroides spp), are isolated from severe infections [9]. This study aimed to determine the type of infecting microorganisms isolated from patients with DFU in a tertiary hospital in Karachi, Pakistan and to determine the susceptibility of these organisms to antimicrobial drugs.

## Materials and methods

A prospective observational study was conducted at the Civil and Dow University Hospitals of Dow University of Health Sciences, Karachi, Pakistan. One hundred consecutive patients with diabetes aged ≥ 18 years who presented with foot ulcers from June to December 2016 were included in this study. Patients with end-stage renal failure requiring regular hemodialysis, those with a history of previous vascular surgery on the involved limb, and those who received hyperbaric oxygen therapy or maggot therapy were excluded from this study. Patients with unrelated skin diseases around the involved foot were also excluded. All patients provided formal informed consent to participate in the study.

Patient characteristics, the results of clinical examinations, and the details of each DFU were recorded. The latter included the anatomical site of each DFU, whether the dorsal or plantar aspect of the foot was predominantly involved, the Wagner Grade (Grades 0 to 5) of the ulcer, peripheral pulses, and any sensory deficits. If ulcers involved the toes, the individual toes were recorded. Specimens for swab culture were obtained after washing each ulcer with saline and applying a sterile cotton-tipped swab to the base of the ulcer for five to 10 seconds. The swab was immediately immersed in transport medium and sent to the microbiology laboratory. Tissue samples were obtained, after washing and debriding the ulcer, by scraping the ulcer base or the edges of the wound with a sterile curette. These samples were stored in sterile containers before being transported to the microbiology laboratory. Antimicrobial susceptibility tests were performed using the disc diffusion technique, as recommended by the guidelines of the Clinical and Laboratory Standards Institute. Due to the non-availability of facilities, Gram-negative bacilli were not tested for the presence of extended-spectrum β-lactamase.

## Results

A total of 100 patients, 62 men and 38 women, were included in this study. DFUs were present in the right and left feet of 41 and 59 patients, respectively, with 68 having forefoot ulcers. The duration of diabetes was > 10 years in 84% of patients. Most patients were from the lower middle class, earning less than 100 US dollars per month, and 69% were educated up to the secondary level. Hyperlipidemia, hypertension, and/or ischemic heart disease were present in 65% (Table 1), peripheral neuropathy was present in 68.5%, chronic osteomyelitis in 57%, and Wagner Grade 2 and Grade 3 ulcers in 37% and 49%, respectively (Table 2).

**Table 1:** Comorbidities Associated with Diabetic Foot Ulcers

Associated Medical Problems	Frequency	Percentage
None	1	1%
Smoking alone	4	4%
Obesity alone	2	2%
Hyperlipidemia alone	18	18%
Hypertension (HTN) alone	44	44%
Ischemic heart disease (IHD) alone	3	3%
Smoking + HTN + IHD	12	12%
Smoking + HTN	16	16%

**Table 2:** Severity of Ulcers, As Determined by Wagner Grade, in Patients with Diabetic Foot Ulcer

Grade	0	1	2	3	4	5	Total
Patients (%)	0 (0%)	0 (0%)	37 (37%)	49 (49%)	12 (12%)	2 (2%)	100

Infection was polymicrobial in 83% of patients, with E. coli isolated from 63% and S. aureus from 58%. In 17% of patients, infection was monomicrobial, with E. coli, S. aureus, and Streptococci being the main organisms isolated (Table 3). Polymicrobial infection was associated with long ulcer duration, poor glycemic control, and improper treatment. Of the isolated organisms, 95% were sensitive to meropenem and 81% to linezolid (Tables 4-5).

**Table 3:** Distributions of Microorganism Isolated from Ulcer Samples

Organism	Frequency	Percentage	Frequency as Monomicrobial	Percentage as Monomicrobial	Frequency as Polymicrobial	Percentage as Polymicrobial
Escherichia coli	63	63.0	3	17.6	60	72.3
Staphylococcus aureus	58	58.0	1	5.9	57	68.7
Pseudomonas aeruginosa	37	37.0	0	0	37	44.6
Streptococcus pyogenes	42	42.0	1	5.9	41	49.4
Streptococcus epidermatitis	14	14.0	0	0	14	16.9
Streptococcus pneumoniae	0	0.0	0	0	0	0
Klebsiella pneumoniae	43	43.0	0	0	43	51.8
Proteus mirabilis	31	31.0	2	11.8	29	34.9
Enterococcus	40	40.0	8	20.0	32	80.0

**Table 4:** Antibiotic Sensitivity of Gram-positive Organisms Isolated from Diabetic Foot Ulcers

Antibiotic	Enterococcus	Staphylococcus aureus	Streptococcus pyogenes	Streptococcus epidermis	Streptococcus pneumoniae
Amikacin	72.5%	65.5%	50%	35.7%	64%
Amoxicillin	67.5%	39.7%	28.6%	28.6%	48%
Aztreonam	77.5%	51.7%	42.9%	57.1%	62%
Cefixime	55%	29.3%	16.7%	42.9%	45%
Ceftriaxone	57.5%	29.3%	14.3%	71.4%	45%
Cefuroxime	72.5%	34.5%	21.4%	85.7%	55%
Piperacillin Tazobactam	92.5%	60.3%	52.4%	78.6%	75%
Fucidic acid	42.5%	43.1%	61.9%	50%	46%
Ciprofloxacin	37.5%	43.1%	61.9%	57.1%	50%
Linezolid	85%	82.8%	73.8%	57.1%	81%
Erythromycin	70%	86.2%	88.1%	71.4%	81%
Meropenem	95%	93.1%	90.5%	78.6%	93%
Metronidazole	80%	72.4%	61.9	64.3%	73%

**Table 5:** Antibiotic Sensitivity of Gram-negative Organisms Isolated from Diabetic Foot Ulcers

Antibiotic	Klebsiella	Proteus	Escherichia coli	Pseudomonas aeruginosa	Enterococcus	Staphylococcus aureus	Streptococcus epidermis
Amikacin	62.8%	48.1%	60.3%	45.9%	72.5%	65.5%	35.7%
Aztreonam	58.1%	38.7%	55.6%	43.2%	77.5%	51.7%	57.1%
Cefixime	46.5%	9.7%	39.7%	16.2%	55%	29.3%	42.9%
Ceftriaxone	51.2%	29.0%	39.7%	24.3%	57.5%	29.3%	71.4%
Cefuroxime	60.5%	32.3%	50.8%	24.3%	72.5%	34.5%	85.7%
Piperacillin Tazobactam	79.1%	74.2%	68.3%	62.2%	92.5%	60.3%	78.6%
Fucidic acid	37.2%	51.6%	38.1%	51.4%	42.5%	43.1%	50%
Ciprofloxacin	44.2%	58.1%	49.2%	51.4%	37.5%	43.1%	57.1%
Linezolid	72.1%	54.8%	79.4%	70.3%	85%	82.8%	57.1%
Erythromycin	65.1%	58.1%	68.3%	78.4%	70%	86.2%	71.4%
Meropenem	93%	93.5%	90.5%	86.5%	95%	93.1%	78.6%
Metronidazole	67.4%	61.3%	71.4%	62.2%	80%	72.4%	64.3%

## Discussion

The lifetime prevalence of DFU in diabetic patients has been estimated to be 15%, with DFU responsible for about 20% of diabetes-related hospital admissions [10]. If not treated properly, DFU can lead to limb amputation. Peripheral neuropathy was the leading contributor to DFU, being present in 68.5% of our patients. Similarly, other studies have reported peripheral neuropathy rates of 61% [8] and 49% [11]. Our finding that DFU was mainly in the forefoot was similar to previous findings [8, 12]. We also found that 86% of DFUs were of Wagner Grades 2 and 3. In comparison, previous studies reported that 35.1% of ulcers were Wagner Grade 3 and Grade 4 [13], that 100% were of Wagner Grades 3 and 5 [14], and that 73% had Wagner Grade 1 [7]. The high percentage of patients in our study with Wagner Grade 2 and Grade 3 may have been due to poor glycemic control, long disease duration, and/or late presentation at the hospital.

The types of microorganisms isolated varied by the severity of the infection. Polymicrobial organisms were isolated from 83% of patients, including most patients with Wagner Grade 3 and Grade 4 ulcers, whereas single organisms were isolated from patients with mild infections. Previous studies have reported polymicrobial infections in 83% [15] and 75% [16] of patients with DFU, whereas another study reported that monoinfection was more common [11].

In our series, the Gram-negative organism, E. coli, was the most frequently isolated bacterial species (63%), followed by S. aureus (58%). In other series, however, S. aureus was the most frequently isolated [17-19]. Although recent studies reported a predominance of Gram-negative aerobes [6, 11, 13, 20], we found that 90% of micro-organisms, both Gram-positive and Gram-negative, were sensitive to meropenem. We found that 85% of S. aureus isolates were sensitive to linezolid, and 84% of E. coli isolated were sensitive to chloramphenicol. Of the Gram-negative organisms isolated in this study, 62% were susceptible to amikacin, similar to previous findings [14, 16, 20], whereas 67% were resistant to ampicillin in 67% of cases, also reported previously [13, 21]. Anaerobic and fungal infections were not detected in our series.

## Conclusions

In this series, 86% of patients had ulcers of Wagner Grades 2 and 3. This may have been due to their late presentation in a tertiary care hospital. Most infections were polymicrobial, with E. coli being the most common Gram-negative organism. Most isolated Gram-negative and Gram-positive microorganisms were susceptible to meropenem. Serious complications of DFU may be avoided by proper clinical evaluation, strict glycemic control, better patient compliance, education regarding foot care, and aggressive surgical and medical treatment.
